# Reinfusion of peritoneal fluid elevates the level of plasma D‐dimer in patients with early‐onset ovarian hyperstimulation syndrome

**DOI:** 10.1002/rmb2.12563

**Published:** 2024-02-14

**Authors:** Shiori Kumazawa, Kazuki Saito, Nanako Hashido, Rinko Ibi, Tomonori Ishikawa, Akira Wakabayashi, Naoyuki Miyasaka

**Affiliations:** ^1^ Department of Comprehensive Reproductive Medicine, Graduate School Tokyo Medical and Dental University Tokyo Japan; ^2^ Department of Perinatal and Maternal Medicine (Ibaraki), Graduate School Tokyo Medical and Dental University Tokyo Japan; ^3^ Department of Obstetrics and Gynecology Tokyo Metropolitan Hiroo Hospital Tokyo Japan

**Keywords:** D‐dimer, ovarian hyperstimulation syndrome, peritoneal effusion, VEGF, venous thromboembolism

## Abstract

**Purpose:**

This study aimed to elucidate the factors that affect the dynamics of blood D‐dimer in ovarian hyperstimulation syndrome (OHSS).

**Methods:**

We retrospectively reviewed medical records from two hospitals and extracted data obtained during assisted reproductive technology and OHSS treatment. Blood D‐dimer levels during hospitalization were plotted against body weight. Other factors possibly related to blood D‐dimer levels were also analyzed.

**Results:**

The analysis included 10 patients with OHSS admitted between January 2013 and June 2023. In all patients, blood D‐dimer levels increased significantly when they convalesced from OHSS and lost weight. None of the patients showed clinical signs of thrombosis, which was confirmed using imaging tests in 8 of 10 patients. Two patients underwent cell‐free and concentrated ascites reinfusion therapy (CART), and their blood D‐dimer levels increased dramatically after the procedure.

**Conclusion:**

Weight change and CART are associated with blood D‐dimer dynamics in OHSS. Our results show that elevated blood D‐dimer levels in patients with OHSS do not always represent the presence of thrombosis. Reinfusion of pooled D‐dimer in ascites may explain the D‐dimer surge during the recovery phase or after CART in these patients. Our study provides new perspectives on the clinical implications of D‐dimer during OHSS.

## Graphical abstract

During the recovery phase of OHSS, blood level D‐dimer increases without thrombosis. Reinfusion of pool D‐dimer in ascites into the vasculature possibly explains this transient D‐dimer rise.

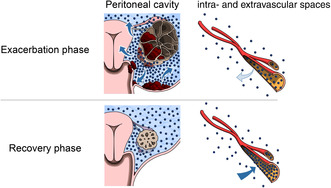

## 1 INTRODUCTION

Ovarian hyperstimulation syndrome (OHSS) is a life‐threatening complication of assisted reproduction.^1^ Gonadotropin preparations for controlled ovarian stimulation induce multifollicular development to obtain multiple oocytes. Vascular endothelial growth factor (VEGF) secreted from the corpus luteum after oocyte retrieval increases capillary permeability, which leads to peritoneal and pleural effusion.^2^ Early‐onset OHSS appears within 10 days after oocyte maturation is triggered, and its pathophysiological condition is self‐limited when no pregnancy occurs, whereas late‐onset OHSS appears more than 10 days after oocyte retrieval and accompanies pregnancy.^2^ As the corpus luteum regresses over time, peritoneal effusion spontaneously ceases, and the fluid in the peritoneal cavity flows back into the blood vessel. Consequently, excess fluid is removed through urination, and body weight gradually decreases. However, hemoconcentration due to drastic fluid shifts can sometimes lead to arteriovenous thrombosis in severe cases.^3^ Hence, managing water balance and preventing arterial or venous thromboembolism are essential in OHSS. These cases require multidisciplinary therapies, such as rehydration, VEGF‐targeted therapy, anti‐thrombogenic therapy, and the use of aspirin.^1^, ^2^, ^3^, ^4^, ^5^

D‐dimer refers to multiple peptide fragments derived from plasmin‐mediated degradation of cross‐linked fibrin‐degradation products during fibrinolysis.^6^ D‐dimer is mainly eliminated through renal clearance and the reticuloendothelial system.^6^ Compared with the thrombin‐antithrombin complex or prothrombin fragments, D‐dimer has a longer half‐life of 8 hours.^7^ Because of its prolonged stability and half‐life, D‐dimers are used as an eminent biomarker for diagnosing deep venous thrombosis or pulmonary embolism.^8^ Owing to its high sensitivity, a negative result practically rules out thrombosis. However, positive results do not directly suggest the presence of thrombosis because several other conditions are associated with elevated blood D‐dimer, such as surgery and pregnancy.^9^, ^10^ Postoperative patients show elevated plasma D‐dimer levels because of hematoma due to the surgical procedure.^9^ For the same reason, after controlled ovarian stimulation and oocyte retrieval, patients seem to show elevated plasma D‐dimer levels due to hematoma in their ovaries and peritoneal cavity.^11^ While thrombosis is one of the most critical complications of OHSS, the credibility of plasma D‐dimer for diagnosing thrombosis in patients with OHSS is not well established. In addition, the implication of drastic fluid shift between the intra‐ and extravascular spaces on plasma D‐dimer levels remains elusive. Herein, we analyzed 10 cases of early‐onset OHSS and reported the dynamics of serum D‐dimer from the exacerbation to the recovery phase of OHSS.

## 2 MATERIALS AND METHODS

### 2.1 Ethical approvals

This study was approved by the Institutional Review Board Committee of Tokyo Medical and Dental University (M2023‐067). As only anonymized medical record information was used, this study is an exception to obtaining informed consent from patients.

We retrospectively reviewed the medical records at Tokyo Medical and Dental University Hospital and Tokyo Metropolitan Hiroo Hospital and extracted data pertaining to patients who were admitted to the hospital due to OHSS from January 2013 to May 2023. Patients whose serum D‐dimer level and body weight were measured less than three times during hospitalization were excluded from the analysis.

Clinical data such as age, height, weight, IVF treatment, blood and image test results, and medication were obtained. Specifically, risk factors for OHSS such as body mass index, history of ovulation disorder, blood level of AMH, total amount of gonadotropin used, and trigger for maturation were also investigated. Because body weight is a validated marker of fluid accumulation, we defined the clinical course of OHSS according to the increase and decrease in body weight; the exacerbation phase stands for gaining body weight, and the recovery phase means a reduction in body weight. Additionally, we availed abdominal circumference as another surrogate marker for ascitic fluid accumulation. We also investigated the influence of cell‐free and concentrated ascites reinfusion therapy (CART) on the blood level of D‐dimer where it is used.

Subsequently, we plotted the body weight, D‐dimer level, white blood cell count, and hematocrit value against the number of days of hospitalization for each patient. In addition, the trend of abdominal circumference and urine volume was also investigated. The interrelationships between these factors over time were visually analyzed, and the implication of CART on body weight and D‐dimer level was also investigated. The blood D‐dimer level was analyzed by immunonephelometry, using Nanopia D‐dimer (Sekisui Medical Corporation, Tokyo, Japan) as the reagent for cases 6, 8, and 10, and LIAS AUTO D‐dimer NEO (Sysmex Corporation, Hyogo, Japan) as the reagent for the other cases.

## 3 RESULTS

From January 2013 to May 2023, 10 patients were admitted to our hospitals due to OHSS. The patient characteristics are described in Table 1. Most of the patients had multiple risk factors for OHSS. Six patients were less than 35 years old. The patients were relatively lean, with nine of the ten patients weighing less than 60 kg at admission. Four patients were previously diagnosed with polycystic ovarian syndrome, and two patients had hypogonadotropic hypogonadism. Blood levels of AMH were 5.0 ng/mL or more in all the patients whose data were available. All patients underwent controlled ovarian stimulation and oocyte retrieval, and nine of them used human chorionic gonadotropin as a trigger for oocyte maturation. In most cases, more than 20 oocytes were collected. All the embryos were frozen, and no fresh embryos were transferred in all cases. On average, patients were admitted to the hospital 3.4 days after oocyte retrieval. Only two patients, cases 9 and 10, underwent CART due to severe abdominal distension and decreased urine volume, which continued after rehydration.

**TABLE 1:** Clinical Characteristics of the Patients.

Characteristic	Case 1	Case 2	Case 3	Case 4	Case 5	Case 6	Case 7	Case 8	Case 9	Case 10
Age (yr)	31	29	38	38	35	33	34	27	35	29
Height (cm)^a^	168	156	157	161	161	168	152	160	160	165
Weight (kg)^a^	56.8	51.0	69.5	50.3	62.3	56.6	51.1	50.8	53.8	50.9
Body mass index	20.1	21.0	28.2	19.4	24.0	20.1	22.1	19.8	21.0	18.7
History of ovulation disorder	PCOS	PCOS	Hypo‐hypo	NA	NA	NA	Hypo‐hypo	PCOS	−	PCOS
AMH (ng/mL)	17.3	NA	12.6	NA	NA	NA	14.3	11.1	5.0	NA
Protocol for COS^b^	PPOS	Short protocol	FSH	Antagonist protocol	Antagonist protocol	CC‐hMG	hMG/rFSH	PPOS	Random start ovarian stimulation	Short protocol
Total amount of gonadotropin (IU)	1500	NA	3075	1350	NA	1200	3000	1800	2925	66 μg^c^
Trigger for oocyte maturation										
hCG	−	+	+	+	+	+	+	+	+	+
Buserelin	+	−	−	−	−	−	−	+	−	−
Number of oocytes picked up	27	23	12	46	NA	37	25	31	27	52
Peak estradiol level (pg/mL)	14 425	7364	3883	8949	6326	>3000	2853	>3000	1470	>3000
Days from oocyte retrieval to hospitalization	6	6	2	3	5	4	1	2	1	4
Clinical presentation of the patients at admission severity of OHSS^d^	Critical	Severe	Severe	Severe	Critical	Severe	Severe	Severe	Severe	Severe
Maximum diameter of ovaries (mm)										
Right ovary	119	90	111	81	87	102	101	81	122	126
Left ovary	74	80	104	77	86	60	105	78	112	86
Ascitic fluid	+	+	+	+	+	+	+	+	+	+
Pleural effusion	+	−	−	−	+	−	−	−	−	−
White blood cell (/μL)	11 800	13 000	11 100	9600	21 200	11 300	7500	13 200	11 700	22 100
Hematocrit (%)	41.1	44.0	45.9	43.1	49.1	43.2	47.0	37.2	34.3	53.0
Total protein (g/dL)	4.6	6.2	6.8	6.9	5.7	5.8	6.6	6.1	3.9	5.2
Albmin (g/dL)	2.7	3.6	3.7	4.0	2.7	3.5	3.6	3.4	2.2	2.8
Medication for OHSS										
Anticoagulant agents	Heparin	Heparin	Heparin	Heparin	Heparin	−	−	Heparin	Heparin	Heparin
Antiplatelet treatment	Aspirin	Aspirin	−	−	−	−	Aspirin	Aspirin	Aspirin	Aspirin
Cabergoline	+	+	−	+	+	−	+	+	+	+
GnRH antagonist	+	−	−	−	−	−	+	−	+	−
Aromatase inhibitor	+	−	+	−	−	−	+	+	+	−
CART	−	−	−	−	−	−	−	−	+	+

Abbreviations: CART, cell‐free and concentrated ascites reinfusion therapy; CC, clomiphene citrate; COS, controlled ovarian stimulation; Hypo‐hypo, hypogonadotropic hypogonadism; NA, not available; OHSS, ovarian hyperstimulation syndrome; PCOS, polycystic ovarian syndrome; PPOS, progestin‐primed ovarian stimulation.

^a^ Height and weight are data at admission.

^b^ Controlled ovarian stimulations were either of the following; PPOS, short protocol, antagonist protocol, random start ovarian stimulation with aromatase inhibitor and hMG, and controlled ovarian stimulation with FSH/hMG with or without CC.

^c^ Case 10 used follitropin delta for COS.

^d^ Severity of OHSS was determined according to the guideline released by ASRM (*Fertil Steril* 2016;106:1634–47).

**FIGURE 1:**
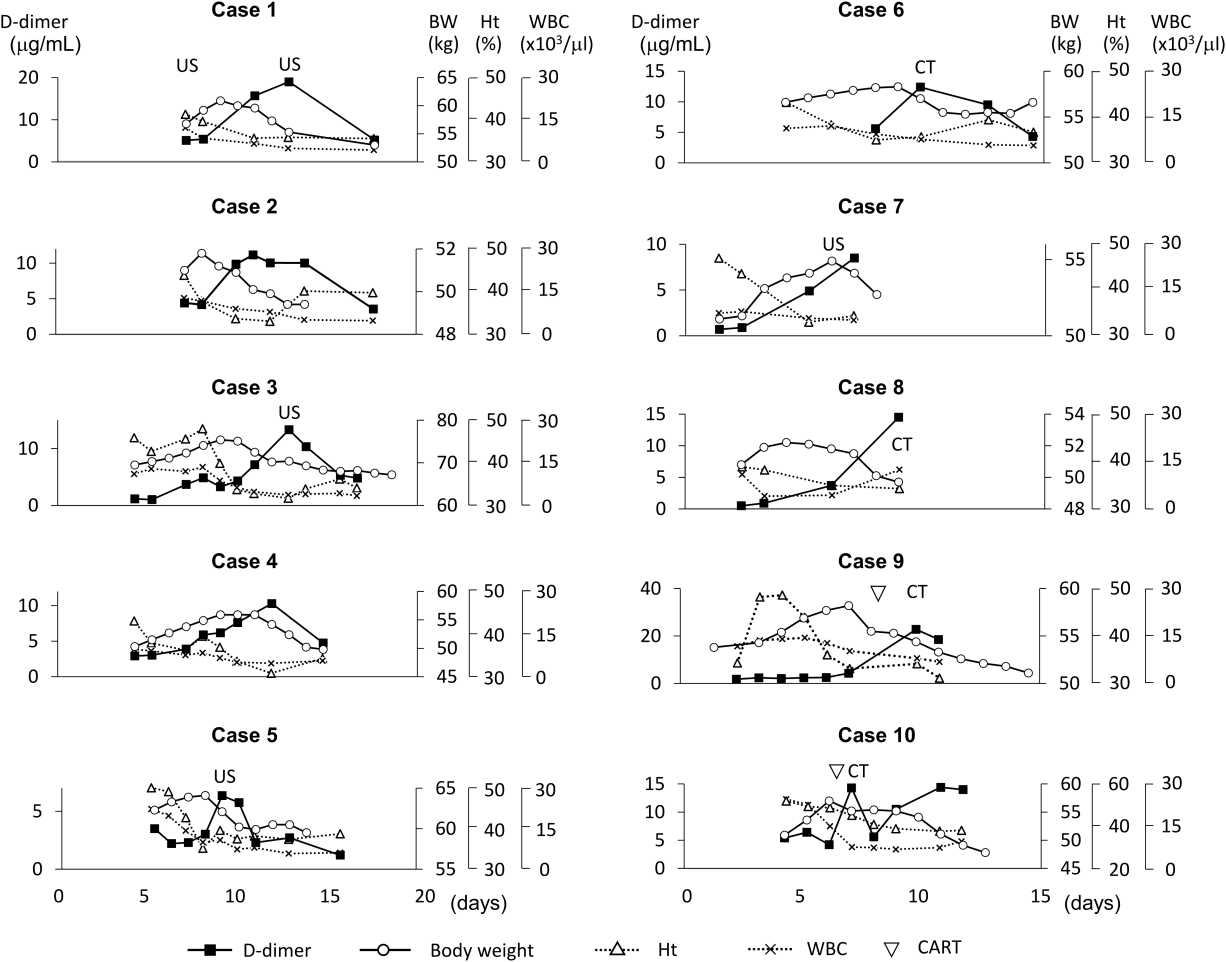
Body weight, blood D‐dimer level, white blood cell count, and hematocrit during hospitalization. Blood D‐dimer levels (black square with solid line) increase when body weight (white circle with solid line) shifts from an increase to a decrease. CART (white arrowhead) was carried out in cases 9 and 10. CT or ultrasonography was performed to investigate the thrombosis at the indicated timing. No case was complicated with thrombosis. Hematocrit value (white triangle with dotted line) and white blood cell counts (cross mark with dotted line) were highest at admission to the hospital and gradually decreased after treatment in most cases. BW, body weight; CART, cell‐free and concentrated ascites reinfusion therapy; CT, computed tomography; Ht, hematocrit; US, ultrasonography; WBC, white blood cell.

Figure 1 shows each patient's body weight, blood D‐dimer level, white blood cell count, and hematocrit changes during hospitalization. White blood cell counts and hematocrit values were highest at admission to the hospital and gradually decreased after treatments. Notably, body weight started to increase before D‐dimer levels increased in all cases. This is with the exception of case 10, in which the D‐dimer level showed a rapid rise when body weight gain stopped and decreased. In case 10, body weight decreased slightly, and the D‐dimer level showed a spike in the day following CART. After CART, the body weight decreased, and blood D‐dimer levels increased, as in the other cases. Regarding abdominal circumference, it increased or decreased along with body weight in many cases, and its peak preceded the D‐dimer surge (Figure S1). However, there were some differences in the trend of urine volume between the cases. In all the cases, urine volume began to increase before body weight began to decrease. The urine volume peak preceded the D‐dimer peak in cases 2, 3, 6, and 8, and the two peaks were synchronized in others (Figure S1). No cases showed physical signs of thrombosis, such as Homans' sign and asymmetric edema of the upper and lower extremities. Computed tomography or ultrasonography also confirmed the absence of thrombosis in 8 of the 10 cases. All patients were discharged from the hospital without any complications, including deep vein thrombosis or pulmonary embolism.

## 4 DISCUSSION

This study provides a novel insight into blood D‐dimer levels in patients with OHSS. Our data showed that reinfusion of peritoneal fluid into the blood vessels raises D‐dimer levels. Since none of our cases were complicated owing to thrombosis, pooled D‐dimer in the peritoneal fluid seemed to flow back to the vessels and raise the blood D‐dimer level. These results indicate, for the first time, that blood D‐dimer levels in patients were modified by exacerbation and restitution of ascitic accumulation.

While thrombosis is a lethal complication of OHSS, there are some problems in its screening. As patients with OHSS are in hypercoagulable condition and often have hematomas in their ovaries and peritoneal cavity, biomarkers for thrombosis seem to be elevated.^12^ D‐dimer levels were slightly elevated in all of our cases at hospitalization. This slight elevation of D‐dimer can be explained by hematoma after ovulation or oocyte retrieval and increases the false‐positive rate for diagnosing thrombosis. Since there was no pregnant case, the clinical condition of OHSS recovered spontaneously, and body weight returned to the initial state in approximately 2 weeks. Notably, blood D‐dimer levels were conversely increased against the decrease in body weight in all cases. No cases were complicated with thrombosis, and CT or ultrasonography also denied the presence of thrombus in the major vessels in most cases. Therefore, the elevation of D‐dimer levels in the recovery process of OHSS may be the result of something other than thrombosis. In this respect, hyperpermeability caused by OHSS may explain the unique dynamics of D‐dimer levels.

**FIGURE 2:**
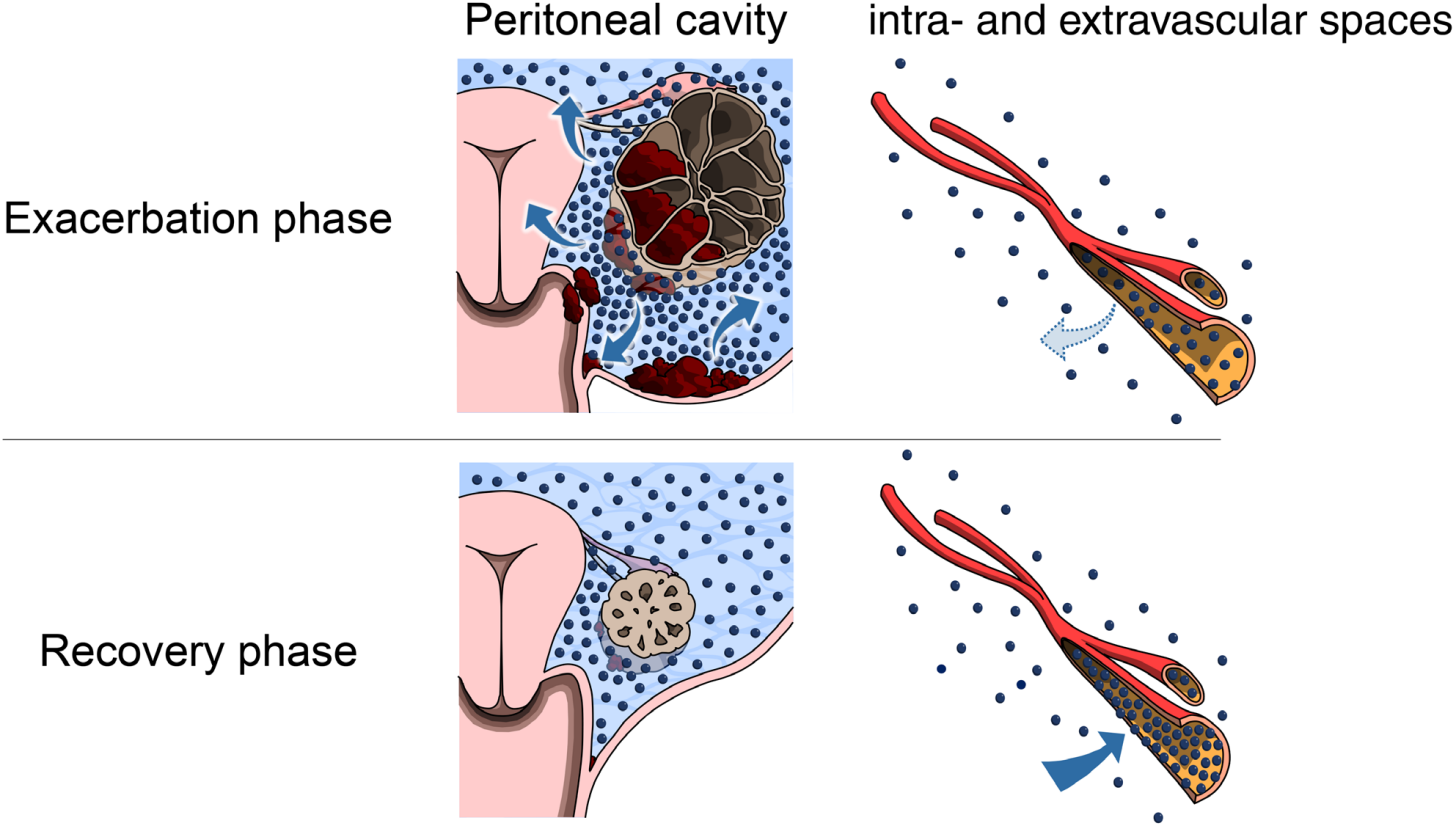
Hypothetical dynamics of D‐dimer between the intra‐ and extravascular spaces. The blue dots represent D‐dimers. During the exacerbation phase, D‐dimer is released from blood clots in the ovary and peritoneal cavity. Because of vascular hyperpermeability, D‐dimer is pooled in the ascitic fluids. During the recovery phase, pooled D‐dimer in ascitic fluid is reinfused into the vasculature.

Vascular hyperpermeability dramatically increases on several occasions, such as in acute and chronic inflammation, cancer, and wound healing.^13^ In OHSS, excessive VEGF secreted from multiple corpus luteum also increases vascular hyperpermeability.^2^ By opening interendothelial junctions and inducing vesiculo‐vacuolar organelles and fenestrations, the capillary wall sometimes allows even red blood cells to pass through it.^14^ Considering that D‐dimer is the smallest product of fibrin degradation, with a molecular weight of 190 kDa,^15^ D‐dimer passes through capillary walls easily when its permeability is increased. On the contrary, the CART system removes cell components and concentrates proteins such as albumin and globulin using a plasma‐separating membrane.^16^ The molecular size of albumin is approximately 66.5 kDa, and that of immunoglobulins is approximately 160 kDa.^17^, ^18^ Hence, the CART system concentrates D‐dimer and reinfuses it into the blood circulation. In fact, elevated D‐dimer levels after CART have been reported in gynecological cancer patients.^19^ Findings in our CART cases are concordant with the result of the previous study and show that the D‐dimer surge after CART is equally applicable to patients with OHSS. In OHSS, ascites are also reinfused spontaneously into the vasculature during recovery. Therefore, we hypothesized that pooled D‐dimer in peritoneal fluid flows back into the circulation in parallel with ascitic reinfusion into the vasculature (Figure 2).

White blood cell counts and hematocrit values were highest at admission and decreased thereafter in most cases (Figure 1). Compared with D‐dimer or other proteins, white and red blood cells are much larger and are retained inside the vasculature. Consequently, the localization of these cells depends little on the fluid shift caused by hyperpermeability, with hemoconcentration developing during the exacerbation phase. Along with rehydration, plasma volume increases, and white blood cell counts and hematocrit value decreases. Urine volume largely depends on the amount of circulating plasma. Generally, as the hyperpermeability decreases, leakage of intravascular fluid reduces, and ascitic fluid flows back into the vasculature. In addition to these factors, the infused fluid volume also determines the urine volume and is the difference between the cases.

While our cases showed an inverse correlation between body weight and blood D‐dimer level during the recovery phase of OHSS, several limitations associated with the present study warrant mention. First, the D‐dimer level in the peritoneal fluid was unknown. However, intraperitoneal blood derived from repeated ovarian puncture or ovulation releases D‐dimer directly into the peritoneal fluid. In addition, considering that the molecular size of D‐dimer is much smaller than the size that passes through the capillary wall under hyperpermeability, intravascular D‐dimer seems to leak out with the peritoneal effusion and flow back to the circulation accompanied by reinfusion of the ascites. Notably, two of our patients with OHSS showed a D‐dimer increase after CART, suggesting that the concentration and reinfusion of ascites to the circulation induce a D‐dimer surge. The increase in D‐dimer is induced by the spontaneous recovery process of OHSS through a dramatic fluid shift from the peritoneal cavity to the intravascular space.

Second, we did not analyze cases where thrombosis occurred. As fluid shift possibly affects D‐dimer levels in patients with and without thrombosis, understanding their association is indispensable. While we showed that D‐dimer levels increase without thrombosis in the recovery phase, D‐dimer levels during the exacerbation phase also deserve attention. For example, in a case complicated owing to thrombosis during the exacerbation phase, D‐dimer may leak out to the extravascular space with peritoneal effusion, and the blood test shows a lower D‐dimer level than expected. These notions warn us of D‐dimer's false‐positive and false‐negative results when screening for thromboembolism in patients with OHSS.

Lastly, body weight was a surrogate marker of ascitic volume, and the genuine association between ascites and blood D‐dimer is still unknown. Due to hyperpermeability, fluid leaks out to the third space, such as the peritoneal and pleural cavities, and tissues throughout the body. Therefore, body weight change represents the water balance of the whole body. Notwithstanding the discrepancy between body weight and ascitic volume, ascitic fluid seems to account for a large proportion of fluid accumulation during OHSS since abdominal circumference, another surrogate maker of ascites, increased or decreased along with body weight (Figure S1). Detailed fluid dynamics and D‐dimer trends in patients with OHSS are worth validating in future studies.

In conclusion, our data indicate that serum D‐dimer levels increase during the recovery phase of OHSS. The rise in D‐dimer levels in this phase is often caused by the reinfusion of pooled D‐dimer in ascites into the blood vessels and does not reflect the presence of thrombosis. The serum D‐dimer level is modified by the fluid shift between vessels and the peritoneal cavity due to the unique pathophysiology of OHSS. Our study provides new perspectives on the clinical implications of D‐dimer during OHSS and avoids repeated blood tests or prolonged hospitalization to check the D‐dimer course.

## FUNDING INFORMATION

There are no funders to report for this submission.

## CONFLICT OF INTEREST STATEMENT

The authors declare no conflicts of interest for this article.

## HUMAN RIGHTS STATEMENTS AND INFORMED CONSENT

All procedures were performed in accordance with the ethical standards of the responsible committee on human experimentation (institutional and national) and with the Helsinki Declaration of 1964 and its later amendments. The study protocol was approved by the Ethics Committee of our hospital (M2023‐067). As only anonymized medical record information was used, this study is an exception to obtaining informed consent from patients.

## Supporting information


Figure S1.
