# Supplementary figures and images for: Reinfusion of peritoneal fluid elevates the level of plasma D‐dimer in patients with early‐onset ovarian hyperstimulation syndrome
Source: Reprod Med Biol. 2024 Feb 14;23(1):e12563. doi: 10.1002/rmb2.12563 (PMC10867380; doi:10.1002/rmb2.12563)

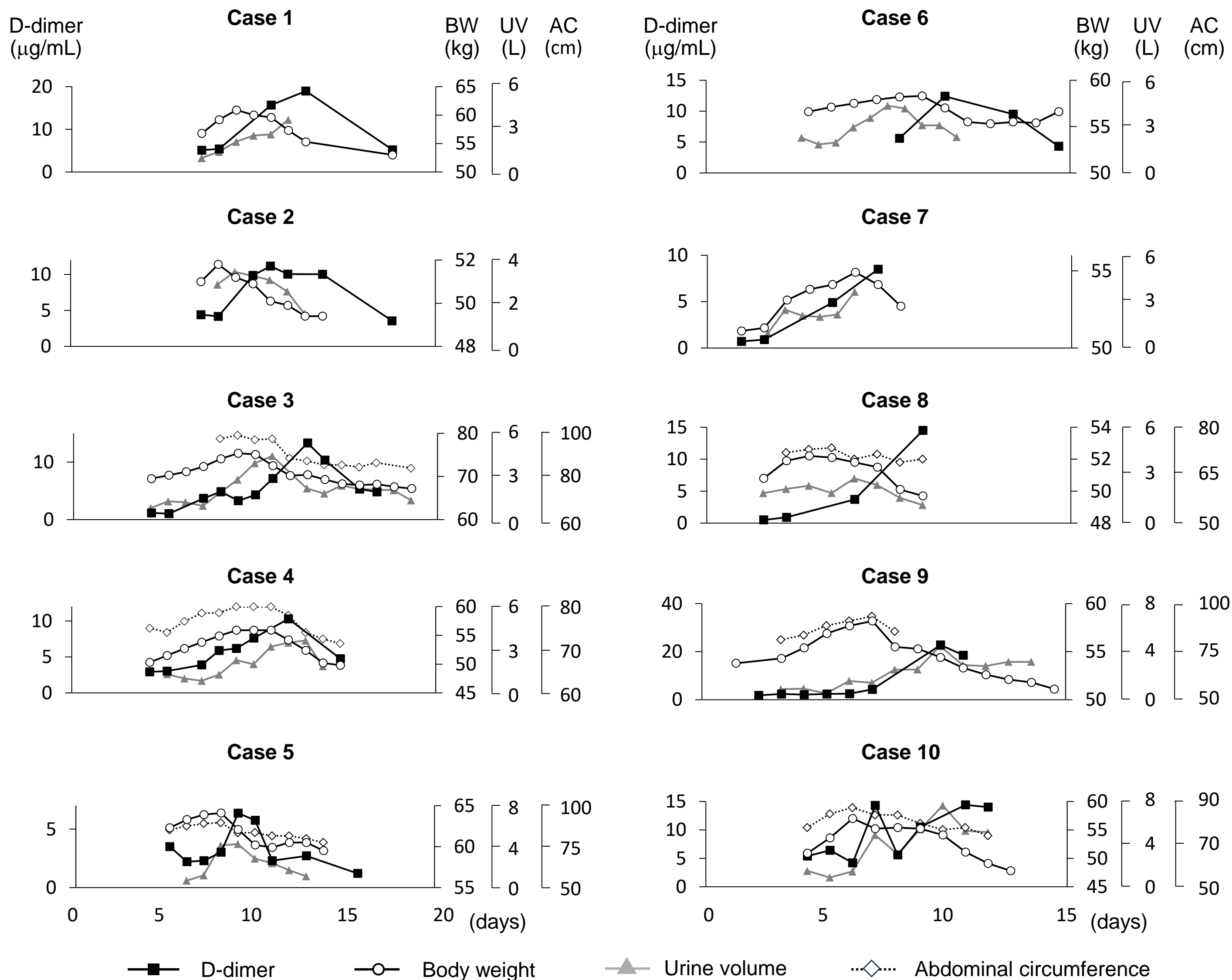

Supplement: Supplementary file 1 — Figure S1. [file RMB2-23-e12563-s001.pdf]
